# Characterization of the Structure, Mechanical Properties and Erosive Resistance of the Laser Cladded Inconel 625-Based Coatings Reinforced by TiC Particles

**DOI:** 10.3390/ma14092225

**Published:** 2021-04-26

**Authors:** Aleksandra Kotarska, Tomasz Poloczek, Damian Janicki

**Affiliations:** Welding Department, Faculty of Mechanical Engineering, Silesian University of Technology, Konarskiego Street 18A, 44-100 Gliwice, Poland; Tomasz.Poloczek@polsl.pl (T.P.); Damian.Janicki@polsl.pl (D.J.)

**Keywords:** Inconel 625, metal matrix composite, laser cladding, erosive wear

## Abstract

The article presents research in the field of laser cladding of metal-matrix composite (MMC) coatings. Nickel-based superalloys show attractive properties including high tensile strength, fatigue resistance, high-temperature corrosion resistance and toughness, which makes them widely used in the industry. Due to the insufficient wear resistance of nickel-based superalloys, many scientists are investigating the possibility of producing nickel-based superalloys matrix composites. For this study, the powder mixtures of Inconel 625 superalloy with 10, 20 and 40 vol.% of TiC particles were used to produce MMC coatings by laser cladding. The titanium carbides were chosen as reinforcing material due to high thermal stability and hardness. The multi-run coatings were tested using penetrant testing, macroscopic and microscopic observations, microhardness measurements and solid particle erosive test according to ASTM G76-04 standard. The TiC particles partially dissolved in the structure during the laser cladding process, which resulted in titanium and carbon enrichment of the matrix and the occurrence of precipitates formation in the structure. The process parameters and coatings chemical composition variation had an influence on coatings average hardness and erosion rates.

## 1. Introduction

Metal-matrix composite (MMC) coatings have been constantly developed in recent times due to the increasing demand and requirements of the industry in the field of surface wear resistance. Production of MMC coatings on machine parts can significantly extend their service life and at the same time reduce costs of the regeneration or replacement of worn parts [1,2,3,4,5]. Nickel-based superalloys show attractive properties including high tensile strength, fatigue resistance, high-temperature corrosion and oxidation resistance in aggressive environments, together with high-temperature toughness and ductility. The combination of these properties makes these alloys widely used in many industries including aerospace, chemical and energy industry [6,7]. In addition, nickel-based superalloys are also used as coatings on machine parts to improve their corrosion resistance. The studies conducted by Abioye et al. [8] and Nemecek et al. [9] show the positive effect of laser cladding of Inconel 625 coatings on the corrosion resistance of the S355 and AISI 304 steel surfaces. The production of composite coatings based on nickel-based superalloys has a high application potential due to the combination of the unique properties of these alloys and the increased wear resistance of the surface. The previously conducted studies [10,11,12,13] show that the production of nickel-based superalloys coatings reinforced with WC, Cr_3_C_2_, VC, TiC, TiB_2_ particles can improve the hardness and wear resistance of the coatings. Taking into account the advantageous properties of the nickel-based superalloys, it is reasonable to select the reinforcing material with high thermal stability in order to ensure high wear resistance in both low and high temperatures. Titanium carbide (TiC) is not only characterized by high hardness (2859–3200 HV) and strength (240–390 MPa), low density (4.92 g/cm^3^), but also has a high melting point (3180 °C) and high thermal stability, which makes it highly attractive for producing nickel-based superalloys matrix composites [14,15,16,17]. The laser cladding process is commonly used for the production of MMC coatings. This technology is characterized by many advantages, including high heating and cooling speeds, which leads to the creation of unique structures, the negligible influence of the base material on the chemical composition and properties of the produced coatings, high precision and efficiency [18,19]. Moreover, laser cladding can be used for producing both in-situ and ex-situ MMC coatings [20,21,22,23].

Research on the production of TiC reinforced nickel-based superalloys coatings using laser cladding technology was previously conducted by Gopinath et al. [24]. The authors used Inconel 718 as coating matrix material and their research was focused on the effect of changing the thermal cycle in the molten pool on the final MMC coating structure. Jiang et al. [25] produced Inconel 625 based coatings reinforced with nano-TiC particles by micro particles insertion and partial dissolution in the structure. As a result, the hardness and modulus of the produced coating increased in comparison to the Inconel 625 coating. Lian et al. [26] tested the dependence of laser cladding of Ni-based coatings reinforced by TiC particles parameters on coatings hardness and wear resistance. Bakkar et al. [27] investigated the high volume percent TiC reinforced (25–70%) Inconel 625 composites. Cao and Gu [28] investigated the microstructure and properties of Inconel 625 matrix composite coatings with 2.5 wt.% TiC nano-particles. The previously conducted researches in this field [24,25,26,27,28] show that nickel-based superalloys composite coatings reinforced by 2.5–50% TiC particles are characterized by 10–45% higher hardness and up to 6% higher wear resistance than metallic nickel-based superalloys coatings. The results [27] also show that the increased TiC particles content of 50–70% may cause defects in the microstructure due to lack of matrix penetration.

The aim of the following research was the production of Inconel 625-based MMC coatings reinforced with 10, 20 and 40 vol.% of TiC using the laser cladding process. The tests were performed to determine the influence of laser cladding process parameters on the structure, hardness and solid particle erosive resistance of the produced MMC coatings in comparison to Inconel 625 metallic coatings.

## 2. Materials and Methods

For the study, the flat surface of the base material (10 mm thick, as-received S355JR low-alloy steel, Cognor, Stalowa Wola, Poland) was prepared by grinding, cleaning and degreasing with ethyl alcohol (Stanlab, Lublin, Poland). For the coating production, Inconel 625 (Metcoclad 625, Oerlikon, Westbury, NY, USA, gas atomized spheroidal powder) and TiC (Goodfellow, Huntington, UK, 50–150 µm, purity 99.8%) powders were used. The chemical composition of the base material and Metcoclad 625 powder are presented in Table 1. Powder mixtures of Metcoclad 625 with the addition of 10, 20 and 40 vol.% TiC were prepared, mixed and dried for 1 h at 50 °C. The laser cladding process was carried out without preheating.

The laser cladding process was perfomed on the stand equipped with a disc laser TRUMPF Trudisc 3302 (TRUMPF, Ditzingen, Germany) (Table 2), a numerically controlled system for positioning the processed material in relation to the laser head and gravitational powder feeder system. For the laser cladding process, the laser beam focus (diameter of 200 μm) was set 30 mm above the base material surface. For the laser cladding process, argon was used as shielding gas (10 L/min) and powder transporting gas (3 L/min). The powder during the laser cladding process was injected directly into the molten pool. To determine the optimal parameters of laser cladding, single-pass coatings were produced with a laser power range of 1400–2300 W, cladding speed of 0.1–0.25 m/min, powder feed rate of 0.03–0.05 g/mm and heat input of 500–560 J/mm. The parameters’ range was chosen based on the previous experience [11,19]. The proceeding analysis of single-pass coating geometry, dilution and TiC particles distribution throughout the volume of the coatings allowed determination of the optimal parameters for producing multi-run coatings (Table 3). The multi-run coatings were produced with a 40% overlap. For each set of parameters, one multi-run coating was prepared.

The research included the penetrant tests for the verification of the presence of the cracks (colour contrast technique, penetrant MR 68 NF, developer MR 70, cleaner MR 79, MR Chemie, Unna, Germany) in the multi-run coatings surfaces, the macrostructure observations of fabricated coatings, the microstructure observations and energy dispersive spectroscopy (EDS) analysis using Scanning Electron Microscope (SEM) Phenom World PRO (Thermo Fisher Scientific, Waltham, MA, USA). The coating’s general chemical composition was estimated based on the 4 cross-sectional surface EDS analysis results (magnification 1000×, accelerating voltage 15 kV) for each coating. For the etching, the mixture of HNO_3_ (Chempur, Piekary Śląskie, Poland), HCl (Chempur, Piekary Śląskie, Poland), acetic acid (Stanlab, Lublin, Poland) and glycerol (Poch, Gliwice, Poland) (etchant 89 according to ASTM E 407-99) was used [29]. The coating’s dilution rate was measured using Equation, where *F_BM_* is the melted cross-sectional area of the substrate and *RA* is the cross-sectional area of reinforcement of the clad. The cross-sectional areas were obtained using AutoCad 2018 software (Autodesk, CA, USA).

$$U=\frac{F_{BM}}{F_{BM}+RA}\times 100\;\left[\%\right]$$

**Figure 1:**
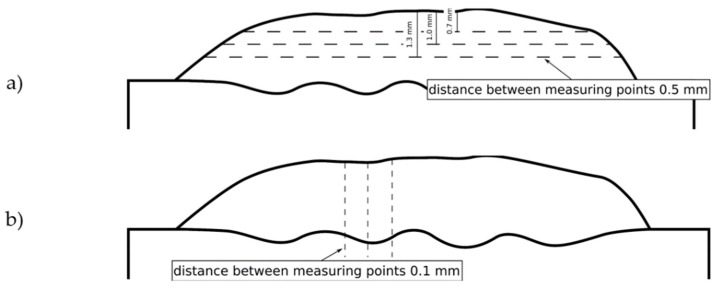
The Vickers microhardness measuring lines scheme, (**a**) measurements across the beads, (**b**) measurements from the coating surface to the base material.

X-ray diffraction (XRD) analysis was proceeded using a PANalytical X’Pert PRO diffraction system (Malvern Panalitycal, Malvern, UK) with filtered radiation from the lamp with a cobalt anode. The X-ray diffraction patterns were recorded from the ground coatings surfaces. The diffraction profiles were obtained in the 2θ range between 25° and 130° in continuous scan mode with a step size of 0.1444°. The counting time per step was 22.695 s. To assess the produced MMC coatings properties, the Vickers microhardness measurements were performed using Wilson 401MVD Vickers microindentation tester (Wilson Instruments, Instron Company, Norwood, MA, USA) and the solid particle erosive tests (device manufactured in Welding Department, Silesian University of Technology, Gliwice, Poland) were carried out according to the ASTM G76-04 standard [30]. The microhardness measurements were performed with a 200 g load and dwell time of 12 s in three lines across the beads at a distance of 0.7, 1.0 and 1.3 mm from the surface (Figure 1a). The distance between consecutive measuring points was 0.5 mm. Additionally, the microhardness measurements were completed in three lines through the coatings from the surface to the base material with the distance between consecutive measuring points of 0.1 mm (Figure 1b). For the test solid particle erosive test, the Al_2_O_3_, 50 µm diameter, abrasive particles in dry air were used as erodent. The velocity of abrasive particles was 70 m/s and its feed rate was 2 g/min. The test lasted for 10 min. The tested sample surface was located at a distance of 10 mm to the nozzle. The test was carried out for each sample with an impingement angle of 90° and 30°. For each angle, three tests were performed. As a result of the solid particle erosive test, mass loss was obtained using a laboratory scale with an accuracy of 0.0001 g. The erosion rate was counted for each sample according to ASTM G76–04 standard [30]. After the erosive tests, the received craters were observed on Scanning Electron Microscope ZEISS SUPRA 35 (ZEISS, Jena, Germany).

## 3. Results and Discussion

**Figure 2:**
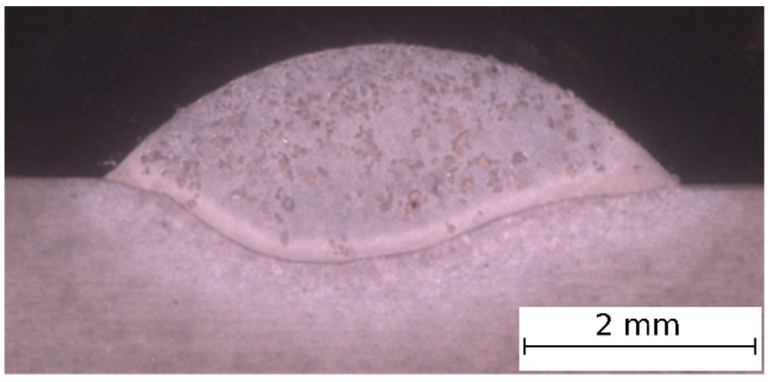
The macrograph of single-pass coating (40% TiC, laser beam power 2100 W, speed 0.25 m/min, powder feed rate 0.05 g/mm).

The macrograph of representative single-pass composite coating is presented in Figure 2. The observations allowed to find that the laser cladded single-pass coating is metallurgically bounded with the base material surface. On the basis of the macroscopic observations of the single-pass coatings, it has been found that the optimal range of laser cladding parameters is very narrow. In the case of the coatings produced with the lowest laser beam power of 1400 W and speed of 0.15 m/min, the insufficient penetration and the TiC particles accumulation on the coatings surface, causing a lack of proper distribution, were observed. The increase in laser beam power to 1850 W, with constant heat input, caused slightly better TiC particles distribution, while in the case of the highest laser beam power 2300 W and speed 0.25 m/min, the penetration and coatings dilution was too high (maximum of 57.2%). The optimal penetration, dilution and reinforcing particles distribution was received for the coatings fabricated by 2100 W power laser beam, with the speed of 0.25 m/min and powder feed rate of 0.04 and 0.05 g/mm.

**Figure 3:**
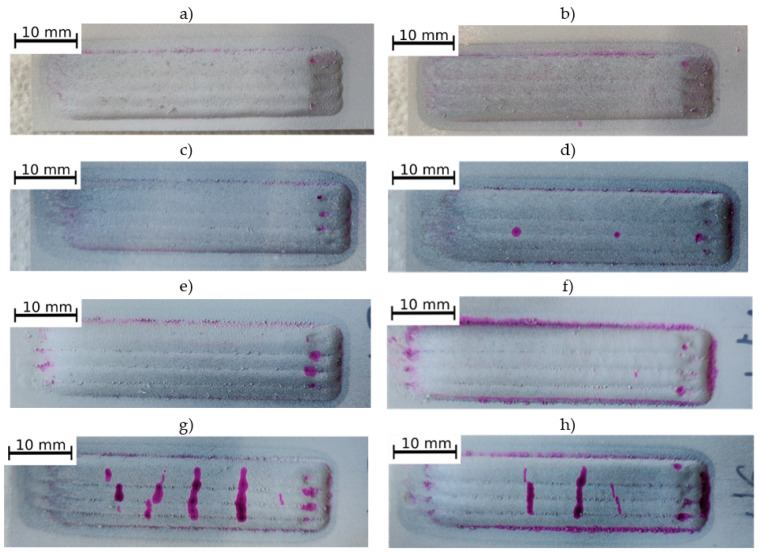
The penetrant test results of laser cladded Inconel 625/TiC coatings (**a**) I-01, (**b**) I-02, (**c**) M-01, (**d**) M-02, (**e**) M-03, (**f**) M-04, (**g**) M-05, (**h**) M-06 (coatings designation according to Table 3).

The surface views after penetrant testing of the produced multi-run coatings are presented in Figure 3. The indications visible around coatings and in the beginning and end areas of the beads resulted from the surface roughness. On the M-02 (Figure 3d) coating can be seen two non-linear indications, which, because of the surface roughness, can be qualified as false indications. The largest linear indications caused by cracks can be observed on the surfaces of coatings M-05 (Figure 3g) and M-06 (Figure 3h), which were produced using a powder mixture with the highest TiC contribution (40 vol.%). The results of this study indicate the negative impact of increased volume fraction of TiC in the Inconel 625 matrix on coating cracking during the laser cladding process with the same parameters. This is due to the increased brittleness of the MMC coating along with the increase in the proportion of the reinforcing phase. As previously investigated [31], the presence of cracks on the surface may deteriorate the erosion resistance.

**Figure 4:**
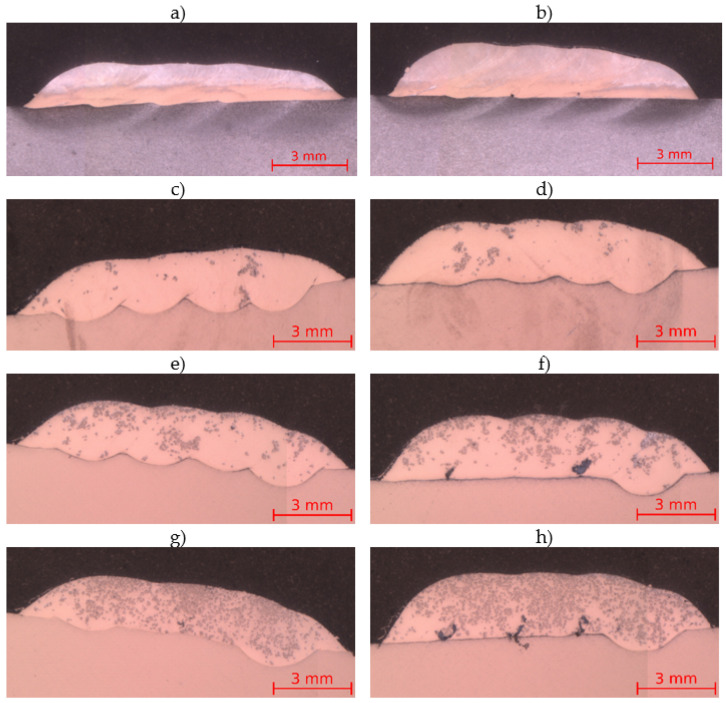
The laser cladded Inconel 625/TiC coatings macrostructures (**a**) I-01, (**b**) I-02, (**c**) M-01, (**d**) M-02, (**e**) M-03, (**f**) M-04, (**g**) M-05, (**h**) M-06 (coatings designation according to Table 3).

The macrographs of produced coatings are presented in Figure 4. The thicknesses, dilutions and measured TiC contents of multi-run laser cladded coatings are summarized in Table 3. The average chemical compositions of coatings cross-sectional regions received from EDS are presented in Table 4. These observations and results allowed to assess the impact of powder feed rate and volume fraction of titanium carbide in the powder mixture on coating thickness, penetration, dilution and uniformity of TiC dispersion in the structure. The results show that together with the increase in powder feed rate, the thickness of the coatings increase. The increased TiC particles content in the powder mixture also caused the increase in the thickness of the coatings fabricated with the same parameters. The measured TiC particles content was in each coating lower than the carbides content in the powder mixture used in the laser cladding process. This phenomenon is directly associated with the fusion and dilution of the coating with the base material, which is causing the volume increase of the coating material. The coatings characterized by higher dilution in each laser cladding parameters set (M-01, M-03, M-05) show a higher decrease in measured volume TiC content (Table 3). At a constant laser beam power of 2100 W and a cladding speed of 0.25 m/min, the use of a lower powder feed rate (0.04 g/mm) resulted in the formation of a higher penetration and dilution of coatings with the same chemical composition. However, the higher powder feed rate (0.05 g/mm) resulted in defects near the fusion line in coatings M-04 (Figure 4f) and M-06 (Figure 4h). In the case of increasing the volume fraction of titanium carbide with the use of constant laser cladding parameters, the penetration and dilution of each composite coating decreased. However, the lowest dilution and penetration were measured for metallic Inconel 625 coatings. The higher penetration can be attributed to the increase in laser radiation absorption level by the presence of TiC particles in the powder mixture, which results in increased heat generation. As a result, the temperature gradient in the molten pool is higher and the mechanism of convective mixing of liquid metal occurs more intensively, leading to higher penetration. On the other hand, the increase of TiC content in the powder mixture leads to Marangoni convection inhibition [32]. As a result, a decrease in penetration can be observed. The coating’s dilution influences the average iron composition, which for composite coatings varies from about 3.3 to 17.98 wt.%. Based on the macroscopic observations, it can be also observed that the powder feed rate change in the tested range does not have a significant effect on the uniformity of titanium carbide dispersion in the structure. Coatings with the lowest volume fraction of titanium carbides show the lowest uniformity of its dispersion in the structure. Titanium carbides accumulated in clusters mainly in the upper part of the coatings with a 10 and 20% volume fraction. It is related to the density of this carbide, which is lower than that of the matrix material. Along with increasing the proportion of titanium carbide to 40%, the homogeneity of the coatings improved.

**Figure 5:**
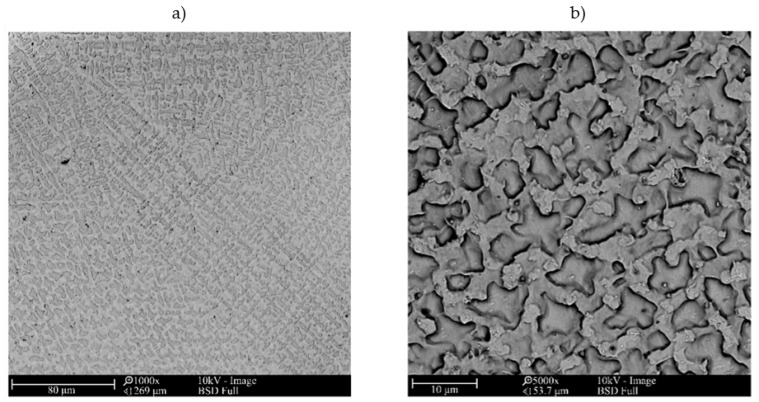
The SEM microstructure of the central bead area of the laser cladded Inconel 625 coating I-01, magnification (**a**) 1000×, (**b**) 5000× (coating designation according to Table 3).

**Figure 6:**
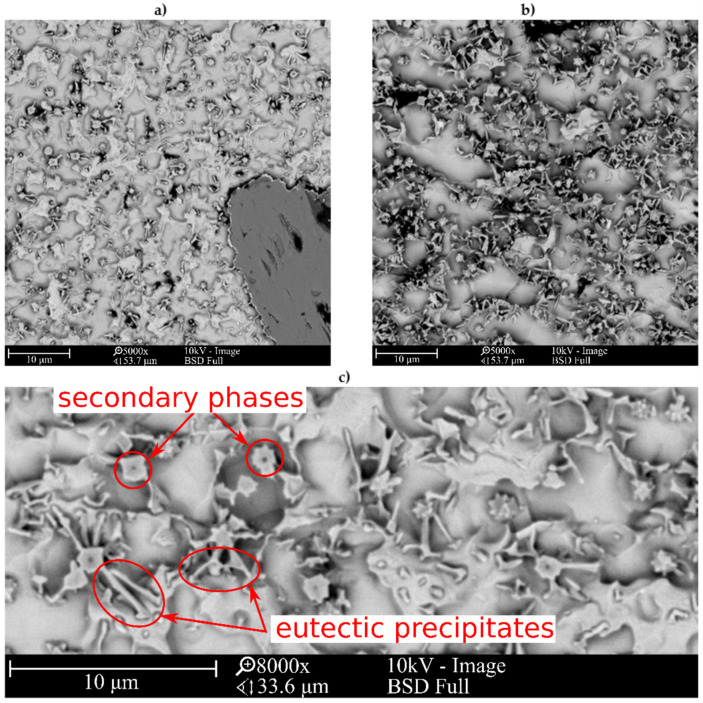
The SEM microstructures of central beads area of laser cladded Inconel 625/TiC coatings; (**a**) M-01 coating, (**b**) M-04 coating, (**c**) M-04 coating (coatings designation according to Table 3).

**Figure 7:**
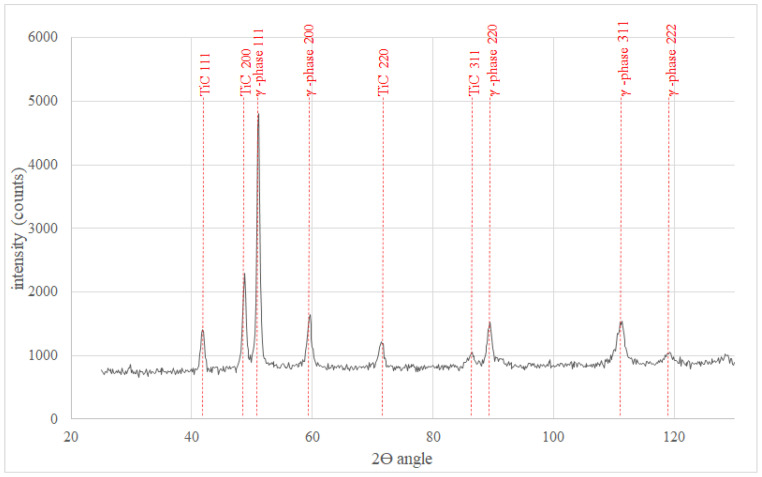
The XRD results of M-05 coating according to Table 3.

**Figure 8:**
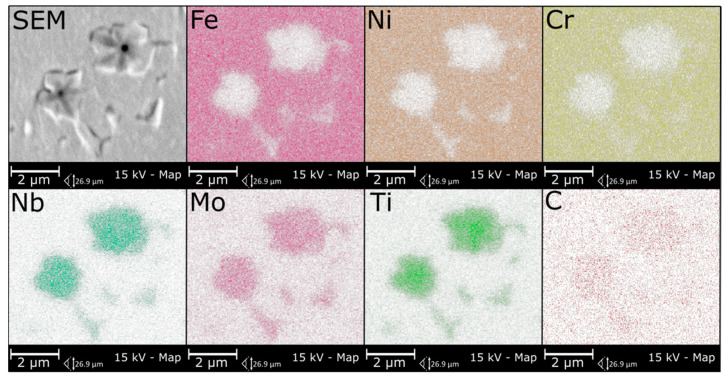
The EDS analysis results of the secondary phases in the matrix, representative coating.

The microstructure of metallic Inconel 625 coatings is presented on Figure 5. The produced composite coating’s microstructure (Figure 6) consists of Inconel 625 matrix and TiC reinforcing particles (RPs). The matrix microstructure consists of austenite dendrites, confirmed by XRD analysis (Figure 7) and minor secondary phases. The austenite dendrites according to EDS analysis consists (Figure 8) mainly of nickel, chromium and iron, while secondary phases are rich in carbon, niobium, molybdenum and titanium. Since carbon and titanium are not present in the Metcoclad 625 powder and are present in the composite coating’s matrix (secondary phases), it can be assumed that the presence of these elements in the matrix is caused by the partial dissolution of titanium carbide RPs. The phenomenon of TiC particles dissolution in Inconel 718 matrix composite coatings depending on molten pool lifetime was investigated in more detail by Gopinath et al. [23]. This is also confirmed by the analysis of the microstructure of metallic Inconel 625 coatings (Figure 5), in which no such precipitates were observed. The metallic Inconel 625 coatings show typical dendritic microstructure with minor constituents in the interdendritic regions, which were previously investigated and reported by Cieslak et al. [33,34]. The columnar dendrites’ growth is caused by temperature gradient and occurs in an opposite to the heat transfer direction.

The secondary phases (Figure 6c) were formed in the structure of the composite coatings as a result of enrichment of the matrix with carbon and titanium and are characterized by blocky and dendritic morphology. As can be observed in Figure 6 and Figure 8, the secondary phases show a gradient distribution of chemical composition. The EDS analysis (Figure 8) revealed that the inner, darker part of these precipitates is rich in titanium. It also allowed to find that Mo and Nb atoms dissolved in the secondary phase’s crystal lattice. On the basis of the XRD and EDS analysis, it can be assumed that the secondary phases are titanium carbides in the crystal lattice, of which the niobium and molybdenum atoms have been dissolved. In the matrix, the microstructure can also be observed minor eutectic precipitates (Figure 6c) formed on secondary phases. This means that the secondary phases formed first during crystallization, and during further cooling, they behave as crystal nucleus for eutectic precipitates between austenite dendrites.

**Figure 9:**
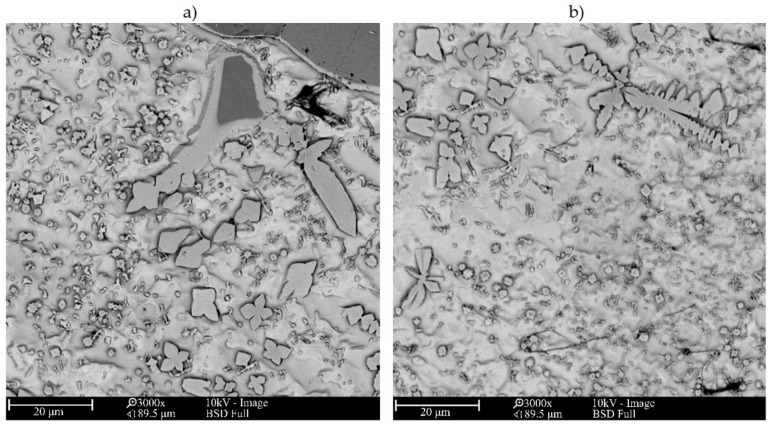
The SEM microstructures of overlap areas of laser cladded Inconel 625/TiC (**a**) M-03 coating, (**b**) M-03 coating (coating designation according to Table 3).

**Figure 10:**
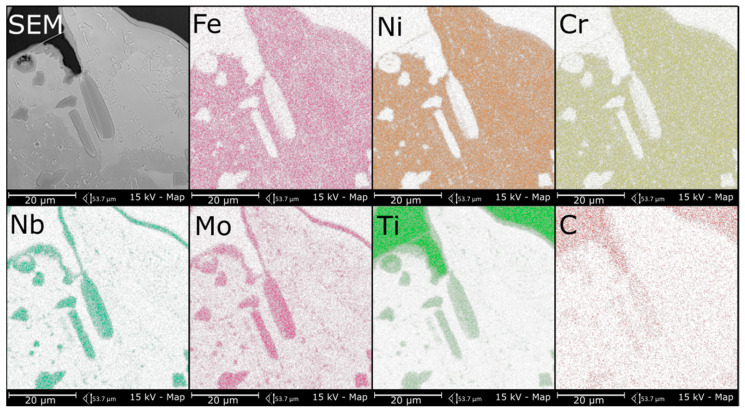
The EDS analysis results of the overlap area, representative coating.

In the overlap area microstructure (Figure 9), as in the case of the central beads area, the austenite dendrites and secondary phases together with minor eutectic precipitates formed on them can be observed. In addition, due to the higher dissolution of RPs in this area, large dendritic precipitates occur. The higher RPs dissolution resulted in liquid metal enrichment in titanium and carbon in this area. Moreover, as can be observed in Figure 9, TiC RPs are more rounded than in the central bead area and the lighter shell formed around RPs. By comparing the overlap area microstructure with the results obtained by Gopinath et al. [24], it can be assumed that the molten pool lifetime was extended in the overlap area in comparison to the central bead area. The composition of shell around RPs and dendritic precipitates formed in overlap area was tested using EDS analysis (Figure 10). The EDS analysis revealed that both shells around RPs and dendritic precipitates are rich in C, Nb, Mo and Ti. The morphology of formed in overlap area dendritic precipitates is characteristic for TiC particles formed in situ [23]. Therefore, during crystallization in the overlap area, dendritic titanium carbides were formed which dissolved niobium and molybdenum atoms in their crystal lattice.

**Figure 11:**
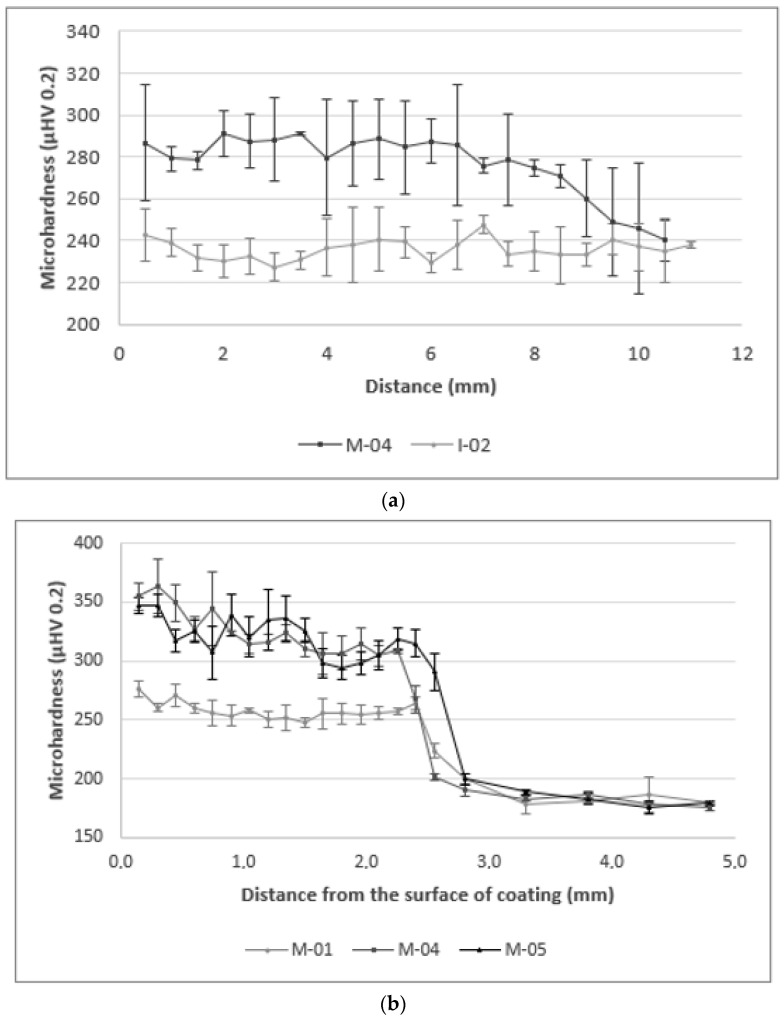
The Vickers microhardness distribution of the coatings; (**a**) across the subsequent beads according to Figure 1a, (**b**) from the surface to the base material according to Figure 1b (coatings designation according to Table 3).

The average Vickers microhardness and erosion rates of produced coatings are presented in Table 5. Figure 11 shows the microhardness distribution of the coatings. The average microhardness of fabricated composite coatings varies from 258 to 342 μHV 0.2. In comparison to metallic Inconel 625 coatings produced with the same laser cladding parameters, the addition of 10 ÷ 40 vol.% of TiC particles to powder mixture led to the average microhardness increase of 5 ÷ 50%. These results are consistent with previous research [25,27]. For composite coatings, the highest average microhardness was measured for M-06 coating with 40 vol.% TiC content, while the lowest average microhardness was measured for M-01 coating with 10 vol.% TiC content. With the increase of TiC content in the structure of coatings fabricated with constant parameters, the average microhardness increased. In the case of coatings with the same chemical composition of the used powder mixture, an increase in the powder feed rate parameter (with a constant power of the laser beam and a constant cladding speed) resulted in an increase in the average microhardness of the coatings, which is associated with lower coating dilution and higher measured TiC particles content. Along with the increase in coatings dilution, the average microhardness of the coatings decreased due to mixing with the base material. The highest values of the standard deviation of microhardness measurements were reported in the case of coatings with the highest content of high hardness titanium carbide. In the case of any of the tested coatings, no significant and repeated changes in hardness were observed in the area of overlapping subsequent beads. The slight decrease in average microhardness towards the end of the measuring lines, which can be observed in Figure 11a, is caused by higher dilution of the first bead, which can be observed on the macrographs. The microhardness distribution from the coatings surface to the base material (Figure 11b) show the highest hardness near the surface and a slight decrease towards the base material. This phenomenon is related to a higher proportion of carbides in the upper area of the coating, due to their lower density than the matrix material, and flowing upwards in the molten metal pool.

**Figure 12:**
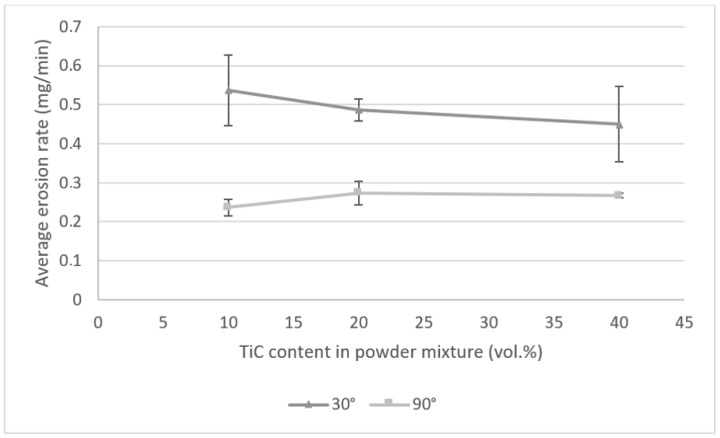
The effect of TiC content in the powder mixture on the average erosion rate of laser cladded composite coatings (laser beam power 2100 W, cladding speed 0.25 m/min, powder feed rate 0.04 g/mm).

**Figure 13:**
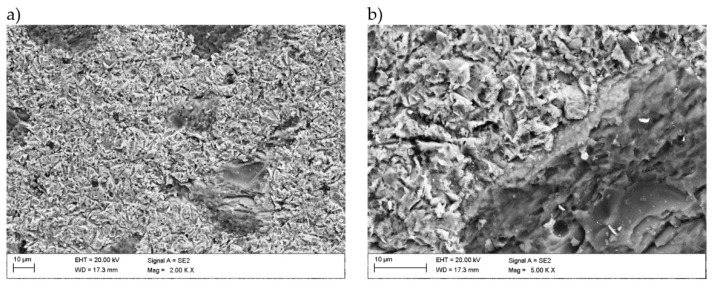
SEM micrographs of craters received after erosion test with impingement angle 90°, magnification (**a**) 2000×, (**b**) 5000×.

The erosion tests showed that for both tested impingement angles, the erosion rates are higher for Inconel 625 metallic coatings than for TiC reinforced composite coatings. Thus, the addition of TiC particles to the powder mixture in 10, 20 and 40 vol.% caused the increase in erosive wear resistance of the Inconel 625 laser cladded coatings. The average erosion rates of all tested coatings with the impingement angle of 30° are higher than the average erosion rates received after the tests carried out with the impingement angle 90°. The dependence of increased erosion wear at a smaller impingement angle (20–30°) compared to the angle of 90° is characteristic for plastic materials [35]. The test results achieved for the 30° impingement angle show that in comparison to the Inconel 625 metallic coating laser cladded with the same parameters, the erosion rate of the TiC reinforced composite coatings decreased by 17 ÷ 37%. In the case of the tests carried out with the impingement angle of 90°, the composite coatings showed 19 ÷ 31% lower erosion rates in comparison to metallic coatings laser cladded with the same parameters. Together with the increase in TiC particle volume content in the powder mixture used for laser cladding of composite coatings, the erosion rate achieved during tests with 30° impingement decreased, while for the tests with 90° impingement angle, the lowest erosion rates were achieved for the powder mixture with 10 vol.% of TiC particles (Figure 12). In this case, with the increase of RPs volume content, the erosion rates slightly increased. It is directly attributed to the increase in the fraction of the TiC phase that is the fraction of brittle material in the coating. Brittle materials are characterized by low erosion resistance at an impingement angle of 90° [35]. Thus, an increase in erosion rate under these conditions with the increase in the TiC fractions is associated with a higher extent of the brittle mechanism of material loss from the eroded surface (Figure 13).

**Figure 14:**
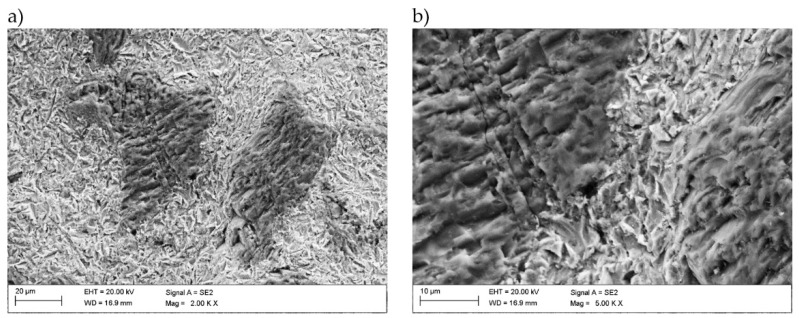
SEM micrographs of craters received after erosion test with impingement angle 30°, magnification (**a**) 2000×, (**b**) 5000×.

SEM observations of craters (Figure 13 and Figure 14) after solid particle erosive tests allowed specification of the erosion mechanism of tested coatings. During proceeded erosion, the plastic deformation occurred on the coating’s matrix surface. On the analyzed micrographs, the coatings matrix and TiC particles can be observed. In the case of surface tested with 30° impingement angle (Figure 14) in the matrix, scars and narrow grooves occur, which proves the plastic deformation and micro-cutting of the material as a result of the interaction with erosive particles. Observations of the surface of titanium carbides show that the mechanism of their erosive destruction is different. On the surface of the TiC particles, sharp edges are visible, which are the result of brittle destruction and detachment of a part of the material during interaction with accelerated erodent particles. Figure 14b also shows a titanium carbide crack. Due to the different properties of TiC, the mechanism of its erosive destruction is brittle.

The SEM micrographs of craters after erosion test with 90° impingement angle are presented in Figure 13. In this case, the matrix of the coatings was also plastically deformed, but the visible grooves are shorter due to a different trajectory of erosive particles. Similarly, the titanium carbides showed a brittle erosive destruction mechanism. In this case, large smooth areas were observed on the surface of titanium carbides, resulting from the fatigue and brittle detachment of a part of the material due to the interaction with erodent particles.

## 4. Conclusions

The research on the production of Inconel 625-based MMC laser cladded coatings reinforced by TiC particles allowed the following conclusions to be drawn:

- The laser cladding process can be used for the production of homogeneous Inconel 625-based MMC coatings reinforced by TiC particles. With a constant laser beam power and cladding speed, along with the increase in powder feed rate, the penetration of base material decreased together with coatings dilution. The powder feed rate has no significant influence on the homogeneity of the coating. The highest homogeneity of the coatings was received using a powder mixture with the highest TiC content (40 vol.%).
- The TiC particles partially dissolved in the structure during the laser cladding process. The enrichment of the matrix in carbon and titanium had an impact on its structure, in which, besides the austenite dendrites, the blocky secondary phases rich in niobium, molybdenum, titanium and carbon appeared. In the overlap area of composite coatings, the increased dissolution of TiC particles occurred, which resulted in the additional formation of dendritic precipitates rich in niobium, molybdenum, titanium and carbon in this area.
- The average microhardness of the composite coatings produced for this research is higher than for the metallic Inconel 625 coatings and varies from 258 to 342 μHV 0.2. The average microhardness increased with the increase in the TiC content in MMC coatings. On the other hand, the increased dilution of the coatings produced with a lower powder feed rate resulted in the average microhardness decrease. No significant and repeated changes in hardness were observed in the area of overlapping subsequent beads.
- In comparison to metallic Inconel 625 laser cladded coatings, the addition of TiC reinforcing particles caused the erosion rates decrease, for both 30° and 90° impingement angles, by 17 ÷ 37% and 19 ÷ 31% respectively. Together with the increase in TiC particle content in MMC coatings, the erosion rate for 30° impingement angle decreased, while for 90° it slightly increased due to the higher content of the brittle phase in the structure. The study allowed definition of the erosive wear mechanism of the coatings. The matrix has been plastically deformed during interaction with the erosive particles, while the TiC particles showed a brittle and fatigue mechanism of erosive wear.

## Figures and Tables

**Table 1:** Chemical composition of S355JR base material and Metcoclad 625 powder.

Material Designation	C	Mn	Si	P	S	Cr	Ni	Mo	Nb	Al	Cu	Fe
	(wt.%)											
S355JR	0.2	1.5	0.2–0.5	max 0.04	max 0.04	max 0.3	max 0.3	-	-	max 0.02	max 0.03	balance
Oerlikon Metcoclad 625	-	-	-	-	-	20.0–23.0	58.0–63.0	8.0–10.0	3.0–5.0	-	-	max 5.0

**Table 2:** Technical specifications of TRUMPH Trudisc 3302 laser.

Property	Value
Wavelength (μm)	1.3
Maximum output power (W)	3300
Laser beam divergence (mm∙rad)	<8.0
Fibre core diameter (μm)	200
Collimator focal length (mm)	200
Focusing lens focal length (mm)	200
Beam spot diameter (μm)	200
Fiber length (m)	20

**Table 3:** Laser cladding parameters.

Designation	Powder TiC Content (vol.%)	Laser Power (W)	Speed (m/min)	Powder Feed Rate (g/mm)	Coating Thickness (mm)	Coating TiC Content (vol.%)	Dilution (%)
I-01	-	2100	0.25	0.04	1.6	-	3.3
I-02	-	2100	0.25	0.05	2.1	-	2.1
M-01	10	2100	0.25	0.04	1.7	8.8	25.5
M-02	10	2100	0.25	0.05	2.1	9.8	12.6
M-03	20	2100	0.25	0.04	1.8	18.3	17.5
M-04	20	2100	0.25	0.05	2.2	19.6	9.8
M-05	40	2100	0.25	0.04	1.9	38.6	14.6
M-06	40	2100	0.25	0.05	2.3	39.7	7.5

**Table 4:** The produced coatings average chemical composition received from EDS surface analysis.

Desigantion	Ni	Cr	Mo	Nb	Fe	Ti
	(wt.%)					
I-01	60.74 ± 1.56	19.82 ± 0.49	10.16 ± 0.79	4.61 ± 0.12	4.67 ± 1.14	-
I-02	63.64 ± 0.63	20.71 ± 0.28	9.47 ± 0.53	4.4 ± 0.58	1.78 ± 0.25	-
M-01	49.13 ± 1.07	16.2 ± 0.41	8.1 ± 0.58	4.7 ± 0.36	17.98 ± 0.95	2.65 ± 0.49
M-02	54.07 ± 4.72	17.59 ± 1.43	9.27 ± 1.09	4.17 ± 0.91	7.89 ± 2.55	3.51 ± 1.05
M-03	50.31 ± 1.28	16.64 ± 0.19	9.73 ± 1.41	3.89 ± 0.34	14.38 ± 0.81	5.37 ± 2.08
M-04	55.13 ± 1.9	18.12 ± 0.5	10.14 ± 1.32	5.1 ± 0.17	5.11 ± 1.58	6.41 ± 1.26
M-05	44.08 ± 2.93	14.11 ± 2.16	7.43 ± 0.87	4.33 ± 0.51	14.48 ± 3.91	14.55 ± 3.75
M-06	47.04 ± 4.94	16.02 ± 1.78	8.59 ± 1.35	4.82 ± 0.76	3.3 ± 1.92	19.73 ± 8.48

**Table 5:** The average Vickers microhardness and erosion rates of Inconel 625/TiC laser cladded coatings (coatings designation according to Table 3).

Designation	Average Microhardness (μHV 0.2)	Average Erosion Rate, (mg/min)	
		30°	90°
I-01	245.5 ± 11.7	0.65 ± 0.07	0.35 ± 0.01
I-02	235.9 ± 9.5	0.68 ± 0.04	0.37 ± 0.04
M-01	257.7 ± 17.6	0.54 ± 0.09	0.24 ± 0.02
M-02	299.1 ± 21.4	0.51 ± 0.07	0.26 ± 0.04
M-03	274.4 ± 18.5	0.49 ± 0.03	0.27 ± 0.03
M-04	313.5 ± 24.7	0.49 ± 0.05	0.30 ± 0.02
M-05	307.1 ± 38.8	0.45 ± 0.10	0.27 ± 0.01
M-06	342.0 ± 38.3	0.43 ± 0.08	0.29 ± 0.02

## Data Availability

Data sharing not applicable.
